# Population dynamics of sporogony for *Plasmodium vivax *parasites from western Thailand developing within three species of colonized *Anopheles *mosquitoes

**DOI:** 10.1186/1475-2875-5-68

**Published:** 2006-08-03

**Authors:** Gabriela E Zollner, Narong Ponsa, Gabriel W Garman, Shreekanta Poudel, Jeffrey A Bell, Jetsumon Sattabongkot, Russell E Coleman, Jefferson A Vaughan

**Affiliations:** 1 Department of Entomology, Walter Reed Army Institute of Research, Silver Spring, MD 20910-7500, USA; 2 Department of Entomology, USAMC-AFRIMS, Bangkok, Thailand; 3 Department of Biology, University of North Dakota, Grand Forks, ND 58202-9019, USA

## Abstract

**Background:**

The population dynamics of *Plasmodium *sporogony within mosquitoes consists of an early phase where parasite abundance decreases during the transition from gametocyte to oocyst, an intermediate phase where parasite abundance remains static as oocysts, and a later phase where parasite abundance increases during the release of progeny sporozoites from oocysts. Sporogonic development is complete when sporozoites invade the mosquito salivary glands. The dynamics and efficiency of this developmental sequence were determined in laboratory strains of *Anopheles dirus, Anopheles minimus *and *Anopheles sawadwongporni *mosquitoes for *Plasmodium vivax *parasites circulating naturally in western Thailand.

**Methods:**

Mosquitoes were fed blood from 20 symptomatic Thai adults *via *membrane feeders. Absolute densities were estimated for macrogametocytes, round stages (= female gametes/zygotes), ookinetes, oocysts, haemolymph sporozoites and salivary gland sporozoites. From these census data, five aspects of population dynamics were analysed; 1) changes in life-stage prevalence during early sporogony, 2) kinetics of life-stage formation, 3) efficiency of life-stage transitions, 4) density relationships between successive life-stages, and 5) parasite aggregation patterns.

**Results:**

There was no difference among the three mosquito species tested in total losses incurred by *P. vivax *populations during early sporogony. Averaged across all infections, parasite populations incurred a 68-fold loss in abundance, with losses of ca. 19-fold, 2-fold and 2-fold at the first (= gametogenesis/fertilization), second (= round stage transformation), and third (= ookinete migration) life-stage transitions, respectively. However, total losses varied widely among infections, ranging from 6-fold to over 2,000-fold loss. Losses during gametogenesis/fertilization accounted for most of this variability, indicating that gametocytes originating from some volunteers were more fertile than those from other volunteers. Although reasons for such variability were not determined, gametocyte fertility was not correlated with blood haematocrit, asexual parasitaemia, gametocyte density or gametocyte sex ratio. Round stages and ookinetes were present in mosquito midguts for up to 48 hours and development was asynchronous. Parasite losses during fertilization and round stage differentiation were more influenced by factors intrinsic to the parasite and/or factors in the blood, whereas ookinete losses were more strongly influenced by mosquito factors. Oocysts released sporozoites on days 12 to 14, but even by day 22 many oocysts were still present on the midgut. The per capita production was estimated to be approximately 500 sporozoites per oocyst and approximately 75% of the sporozoites released into the haemocoel successfully invaded the salivary glands.

**Conclusion:**

The major developmental bottleneck in early sporogony occurred during the transition from macrogametocyte to round stage. Sporozoite invasion into the salivary glands was very efficient. Information on the natural population dynamics of sporogony within malaria-endemic areas may benefit intervention strategies that target early sporogony (*e.g*., transmission blocking vaccines, transgenic mosquitoes).

## Background

Transmission of malaria relies on the successful development of *Plasmodium *parasites within mosquitoes, a process termed sporogony. Sporogony is a complex event involving several morphologically distinct life-stages [1,2] and begins when mosquitoes ingest blood containing male and female gametocytes. Sporogony has three basic phases based on changes that occur in parasite abundance within the mosquito vector. The first phase may be termed "*early sporogony*", a relatively brief period of time where parasites numbers typically decrease within the mosquito. Early events include gametogenesis and fertilization, zygote transformation into ookinetes, ookinete motility through the bloodmeal and peritrophic matrix, penetration across midgut epithelia, and encystment beneath the midgut basal lamina to form oocysts. These events occur during the time that the engorged mosquito is digesting its bloodmeal (*ca*. 2 days). Early sporogony is followed by a period lasting up to a week or more (= "*mid-sporogony*") where parasites are in the oocyst stage. Oocysts grow in size but their numbers remain static. The enlarging oocysts undergo multiple rounds of mitosis to form a syncytium, followed by cellular differentiation to form several thousand daughter cells (= sporozoites). The final phase is "*late sporogony*" which involves release of the sporozoites into the mosquito haemocoel and their subsequent invasion into the mosquito salivary glands. Sporogony is considered complete after sporozoites successfully infect the mosquito salivary glands (ca. 10 to 16 days after initiation) and mosquitoes are able to transmit the parasite to a vertebrate host by infectious bite.

Not every mosquito species supports sporogony of every *Plasmodium *species and disruptions at any point along the developmental sequence diminish the ability of a given mosquito species to transmit malaria. The notion that it is possible to reduce malaria transmission by disrupting sporogony in nature *via *transmission blocking vaccines [3-5] or introduction of refractory genes into vector populations [6-8] has generated new findings that have greatly increased our understanding of the cellular and molecular details of sporogony [9-11]. Sporogony has been less studied in terms of its population dynamics – *i.e.*, quantifying the successive changes in parasite abundance and distribution throughout the developmental sequence [12-21]. When mosquitoes feed on a gametocytaemic person, a portion of the gametocyte population within that person becomes distributed into discrete "sub-populations" (*i.e.*, mosquitoes), somewhat analogous in concept to that of a meta-population. Unless the mosquito feeds on another gametocytaemic person, there is no immigration or emigration of parasites into or out of the mosquito until sporogony is complete. If absolute densities of the various parasite life-stages can be quantified within a mosquito, then the overall efficiency and dynamics of sporogony can be described. While it is virtually impossible to monitor the parasites developing within a single mosquito, the population dynamics of a parasite meta-population can be monitored by sampling many mosquitoes over time provided that all the parasites within a cohort of mosquitoes originated from the same progenitor population (*i.e.*, the same infected person).

Comparing the population dynamics of different parasite populations and parasite species developing within different vector species can reveal the relative efficiencies of the various life-stage transitions. Furthermore, knowledge of transitional efficiencies within naturally-occurring human/*Anopheles *transmission systems can be combined with knowledge of the cellular/molecular processes of sporogony and mosquito immunity to identify which of these processes are the most crucial in regulating plasmodial sporogony in nature. The natural efficiency of sporogony for *Plasmodium *species infecting humans has been described only for the early phase of *Plasmodium falciparum *sporogony in *Anopheles gambiae *mosquitoes at a few localities in tropical Africa [17-21]. This report describes the population dynamics of sporogony for natural *Plasmodium vivax *infections from western Thailand in three indigenous species of colonized *Anopheles *mosquito vectors.

## Methods

### Mosquitoes

Three species of colonized *Anopheles *vectors were used in this study; *Anopheles dirus sensu stricto *(*= dirus *complex), *Anopheles minimus *A (*= minimus *complex), and *Anopheles sawadwongporni *(*= maculatus *complex). These species have been maintained in colony at the Armed Forces Research Institute of Medical Sciences (AFRIMS) in Bangkok, Thailand, for >25, 14, and 9 years, respectively. All three species are important vectors of *P*. *vivax *in Thailand [22].

### Volunteers

Human subjects involved in this study were comprised of 20 adult (≥ 18 yrs) volunteers seeking treatment for uncomplicated malaria at Mae Sot and Mae Kasa clinics, Tak Province in northwestern Thailand. Parasites from 15 of the volunteers were used to estimate the efficiency of early sporogony (*i.e*., gametocyte to oocyst stages). Parasites from five of the volunteers were used to estimate the efficiency of late sporogony (*i.e*., sporozoite production and invasion into salivary glands). Prior to conducting this research, the study protocol received approval from the Institutional Ethics Committee of the Thai Ministry of Public Health and the Human Subjects Research Review Boards of the Walter Reed Army Institute of Research and the University of North Dakota. To diagnose for malaria, thick and thin blood smears were prepared from each volunteer by the clinic staff, stained with 10% Giemsa and examined for malarial parasites. If *P. vivax *gametocytes were present and the volunteers met specific criteria outlined in the approved human subjects use protocols, the volunteers were asked to enroll in the study and complete informed consent forms. Approximately 10 ml of blood was collected by venipuncture and placed in a 37°C water bath. Additional blood samples (fingerprick) were blotted onto strips of filter paper to distinguish the *P. vivax *VK210 and VK247 strains, using nested PCR methods described previously [23]. Volunteers received antimalarial treatment from the clinic staff and were released. Blood smears were later examined more thoroughly (100 microscopic fields at 1,000× oil immersion) to quantify leukocyte densities, asexual and sexual stage parasite densities and gametocyte sex ratios. In this paper, the term "macrogametocyte" is used to describe the female gametocyte detected in peripheral blood smears.

### Infecting mosquitoes

Five to seven day old, con-specific, nulliparous female mosquitoes in cylindrical cardboard containers were transported by automobile from AFRIMS to Mae Sot clinic. Mosquitoes were deprived of sucrose overnight to enhance their willingness to feed. One milliliter of heparinized blood from each volunteer was added to a 5-cm diameter water-jacketed glass membrane feeder fitted with a Baudruche membrane. Blood was kept at a constant 37°C during the mosquito feeding to attract the mosquitoes and prevent premature gametogenesis. Mosquitoes were allowed to feed for 30 minutes and unfed mosquitoes were removed. Engorged mosquitoes were maintained in an insectary at 24°C and ambient humidity for 2 days at the Mae Sot clinic during which time round stages and ookinetes were sampled. In this paper, the term "round stages" is used to describe both unfertilized female gametes (*i.e*., macrogametes) and fertilized zygotes. Mosquitoes were then transported back to the AFRIMS insectary and maintained at 24°C for up to 22 days for oocyst and sporozoite sampling.

### Early sporogony – macrogametocyte sampling

Indirect estimates of macrogametocyte densities per mosquito were obtained using methods similar to those described previously [12]. Macrogametocyte densities per 100 leukocytes were determined for each volunteer from his/her thick blood film. These values were then multiplied by the corresponding leukocyte densities within the mosquito bloodmeals to obtain the estimated density of macrogametocytes in the mosquito bloodmeal. To estimate the average leukocyte densities of the mosquito bloodmeals, the mean volumes of blood ingested by each mosquito species were determined by weighing pools of unfed mosquitoes and pools of mosquitoes immediately after feeding on uninfected blood. The differences in weight between unfed and fed mosquitoes indicated the amount of blood retained in the bloodmeal. A leukocyte density of 7,015 leukocytes per μl blood was used throughout to calculate leukocyte densities. This value was obtained by performing leukocyte counts on 5 of the volunteers and averaging the individual counts. For example, if a microscopic examination of a volunteer's thick smear yielded 10 macrogametocytes per 100 leukocytes and the mean volume of blood ingested by *A. dirus *mosquitoes was 1.5 μl, then the theoretical number of gametocytes ingested would be 10 divided by 100 (= 0.10 macrogametocytes per leukocyte) times 7,015 leukocytes per μl times 1.5 μl blood ingested by *A. dirus*; the product of which yields 1,052 macrogametocytes per mosquito bloodmeal for that hypothetical case.

### Early sporogony – round stage and ookinete sampling and bloodmeal digestion kinetics

**Figure 1:**
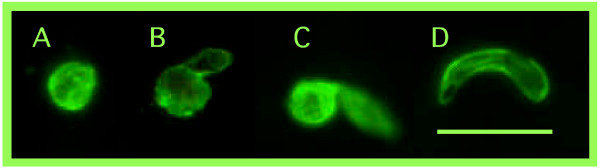
**Early sporogonic life-stages of *Plasmodium vivax *developing within the blood meal of *Anopheles *mosquitoes**. Parasites are visualized by immunostaining with monoclonal antibody specific against the 25 kD protein of sexual stages of *P. vivax*. A. Round stage representing either female gamete or zygote; B. stage II retort ookinete; C. stage III retort ookinete; D. stage IV-VI mature ookinete.

Bloodmeals were examined for round stages and ookinetes using immunofluorescent antibody staining techniques described previously [24]. Groups of mosquitoes were dissected at 12, 18, 20, 22, 24, 26, 36 or 48 h after feeding. The average numbers of mosquitoes dissected per sampling interval for *A. dirus, A. minimus *and *A. sawadwongporni *were 12, 6, and 6 mosquitoes, respectively. Fluorescing parasites were counted using 400X epifluorescent microscopy and classified as either round stages, retort ookinetes (Stages II or III) or mature ookinetes (Stages IV-VI combined) [25] (Figure 1). The kinetics of mosquito bloodmeal digestion was also examined because early sporogony occurs within the context of bloodmeal digestion. *A. dirus *and *A. sawadwongporni *mosquitoes were fed heparinized, uninfected human blood *via *membrane feeding and maintained at 24°C. Bloodmeal volumes were determined as described above. Groups of mosquitoes were dissected at 0, 12, 24, 30, 36 and 48 hours after feeding and individual bloodmeals were diluted 1:200 in buffered saline. Erythrocytes were counted using a haemocytometer and appropriate calculations were made to determine the mean erythrocyte densities of mosquito bloodmeals at each sampling interval.

### Oocyst sampling

Oocysts were counted at various intervals, beginning at 7 to 22 days after infectious feeds. Mosquito midguts were individually excised, placed in a droplet of diluted mercurochrome on a glass slide, compressed with a coverslip and examined at 100–400X bright field microscopy. Oocysts were measured with an ocular micrometer at 400X.

### Late sporogony – sporozoite sampling

For this aspect of the study, only infections that contained high oocyst prevalences were used because the sampling techniques involved were quite meticulous and laborious. From day 7 to 22 after the infectious bloodmeal, groups of 8 to 15 mosquitoes each were removed every other day and processed in such a manner that allowed estimation of the absolute densities of oocysts, haemolymph sporozoites and salivary gland sporozoites within single mosquitoes [12]. First, haemolymph was collected by haemocoel perfusion. The mosquito was immobilized by chilling and restrained by light vacuum drawn through the open end of a bent syringe needle. A small incision was made near the posterior end of the abdomen. A fine-tipped glass needle mounted in a micromanipulator (World Precision Instruments, Sarasota, FL) and connected by tubing to a 500 cc syringe (= hand vacuum/pump) was filled with ca. 15 μl of RPMI media and inserted into the thorax of the restrained mosquito. The haemocoel was flushed by gently pressing the plunger of the syringe. The perfusate exuding from the abdominal incision was collected with a micro-capillary tube and 10 μl was loaded onto a haemocytometer (= haemolymph sporozoite sample). Next, the paired salivary glands were excised and transferred with a fine minuten hook into a glass micro-grinder (Kontes Glass Co., Vineland, NJ) containing 35 μl of RPMI media. Gland pairs were triturated and 10 μl of the triturate was loaded onto the opposite side of the same haemocytometer (= salivary gland sporozoite sample). Both samples were examined at 400x phase-constrast and the sporozoites were counted. Finally, the midgut was dissected and examined for oocysts as described above. Mosquitoes were processed in this manner until a minimum of five infected mosquitoes had been detected for each sampling interval. Only infected mosquitoes were included in data analyses.

### Data analysis

Five aspects of population dynamics were analysed; 1) stage-specific prevalence during early sporogony, 2) stage-specific kinetics of early and late sporogony, 3) estimation of absolute densities and transitional efficiencies between life-stages (*i.e*., cohort life tables), 4) density relationships between life-stages, and 5) aggregation patterns during early sporogony. Chi square analyses were used to compare the prevalences of early sporogonic life-stages among volunteer and mosquito species. Count data on life-stage densities were first tested for normality (Shapiro-Wilk Normality Tests) and the overall effects of independent variables (*e.g*., mosquito species, volunteer number, haematocrit, etc.) on mean life-stage densities were tested using analysis of variance (ANOVA) for normally distributed variables and Kruskal-Wallis ANOVA for non-normally distributed variables. Kinetics of life-stage formation were determined for each infection by plotting over time the mean densities of round stages, and ookinetes (Stages II to VI combined) for early sporogony and the mean densities of oocysts, haemolymph sporozoites and salivary gland sporozoites for late sporogony. When this was done, it was evident that parasite life-stage development was typically asynchronous, with multiple peaks in abundance that overlapped with successive life-stages. To construct cohort life tables, it was necessary to estimate the absolute density entering each life-stage. Estimating density was straightforward for macrogametocytes and oocysts because there was no overlap in recruitment from previous life-stages. Macrogametocyte densities were based on counts from a blood smear, whereas oocysts remained the only life-stage present in mosquitoes for at least a week. However with the more dynamic life-stages (*i.e*., round stages, ookinetes, and haemolymph sporozoites), choosing a single time interval on which to base "peak estimates" for the absolute density of a life-stage was more problematic due to overlapping stage-specific recruitment and multiple peaks in abundance. The ecological literature describes different methods for estimating the numbers entering a life-stage from a series of population samples and each method has its own specific assumptions and requirements [26]. One method that has compatible assumptions with sporogony is to plot life-stage densities over time and compute the area under the curve. Unfortunately, the sampling intervals resulted in truncated density curves (*i.e*., parasite sampling did not begin soon enough or continue long enough to have defined endpoints). Without the tail ends of the curve, the "integration-under-the curve" method yielded inflated estimates of density (data not shown). Instead, a simple ranking system was devised to define the "peak" life-stage densities with which to construct cohort life tables. For each infection and mosquito species, the count data for round stages, ookinetes and sporozoites were ranked from highest to lowest and the arithmetic mean for each data column was designated as the threshold or "cut-off" value. Counts equaling or exceeding the mean were then averaged and the resulting values were used thereafter to represent the "peak" densities for each life-stage within an infection and mosquito species. Counts below the threshold were not used to estimate "peak" life-stage densities. The advantage of this threshold method is that ranking all parasite counts disregarded the time intervals from which a sample was collected. This eliminated temporal variation in the waxing and waning of populations among infections, as well as any developmental asynchrony and bimodal peaks in abundance. A potential disadvantage of this method is that individual mosquitoes within a cohort with inherently low peak densities may have been underrepresented in the construction of life tables. However, this may be mitigated somewhat, because any potential under-representation was applied evenly across all life-stages (except oocysts) within a cohort. Furthermore, multiple parasite cohorts (*i.e*., infections) consisting of a wide range of starting parasite densities were sampled. Once peak estimates of life-stage densities were obtained using the described "threshold ranking" system, the transitional efficiencies of early sporogonic life-stages were calculated using the population mortality coefficient, *k*, or "killing power", which is simply the difference between the peak densities of 2 consecutive life-stages expressed as logarithms [27]. The first major life-stage transition is the macrogametocyte-to-round stage transition, or *k-1*. Thus, *k-1 *= log_10_(macrogametocyte) minus log_10_(round stage) and represents the intensity of parasite losses during female gametogenesis and/or fertilization. The second major transition is the round stage-to-ookinete transition, or *k-2*. Thus, *k-2 *= log_10_(round stage) minus log_10_(ookinete) and represents the intensity of parasite losses during round stage transformation to ookinetes. The third major transition is the ookinete-to-oocyst transition, or *k-3*. Thus, *k-3 *= log_10_(ookinete) minus log_10_(oocyst) and represents the intensity of parasite losses due to ookinetes crossing the midgut and forming oocysts. The total mortality from macrogametocyte to oocyst, or *K*, was calculated by summing the individual k-values (= *k-1+k-2+k-3*). In instances, where a negative *k*-value was computed – signifying a net *gain *in numbers – the value was set to zero because macrogametocytes, round stages and ookinetes are non-replicating life-stages. Mortality coefficients are based on logarithms and thus are not necessarily intuitive to everyone. To make them more intuitive, parasite mortalities can also be expressed as the antilog of *k *values (= "fold loss") or as a "percentage loss", using the formula 100 – 100 (1/antilog k) [17]. Approximately 60 to 70 mosquitoes of each species were required from each infectious feed to acquire a complete life table (*i.e.*, macrogametocyte, round stage, ookinete and oocyst density estimates). Unfortunately, colony production and feeding success differed among the 3 mosquito species and hence, the numbers of complete life tables available for analyses differed among mosquito species. Due to its superior productivity in colony and willingness to engorge from a membrane feeder, *A. dirus *mosquitoes yielded complete life tables from all 15 volunteers. *A. minimus *and *A. sawadwongporni *yielded seven and three complete life tables, respectively, and nine and seven partial life tables (*i.e.*, macrogametocyte, round stage and ookinete estimates only), respectively. Of the 15 volunteer feeds, 10 were "co-infections" – *i.e*., blood from the same volunteer was fed to more than one species of mosquito. Life-stage densities and mortality coefficients from paired data (*i.e*., co-infections) were compared using paired t-tests or Wilcoxon-signed rank tests, depending on whether or not data were normally distributed. Linear regressions were used to determine density relationships among successive life-stages and life-stage mortalities. Significances of density relationships were tested using F-tests. For each infection, stage-specific parasite populations during early sporogony were examined to determine whether they had regular, random or dispersed (*i.e*., aggregated) distributions among mosquitoes. The degree of aggregation or dispersion displayed by a parasite population is indicative of the degree of heterogeneity in mosquito-to-mosquito susceptibility to infection. Green's index of dispersion [28] was computed on entire, unranked data for each infection having more than one infected mosquito per life-stage. Indices of dispersion could not be computed for macrogametocyte populations because macrogametocyte density estimates were obtained indirectly (see above) and did not have sample variances associated with them. Green's index was calculated as: (*s*^2^*/m – 1*)/*(∑x-1*) where *s*^2 ^= variance, *m *= mean, *∑x *= sum. Values can range from *-1/∑(x-1*) indicative of a totally uniform distribution, *'0' *indicative of a totally random distribution to *'1' *indicative of perfect clumping. Green's index is independent of changes in the sample mean and sample size, making it appropriate for comparing different populations that vary in these parameters [29]. Paired t-tests were used to determine significant differences in overall Green's indices for life-stages. A 0.05 level of significance was used for all statistical tests (Statistix v. 8, Tallahassee, FL).

## Results

### Data set

For studies on early sporogony, the number of infections for *A. dirus*, *A. minimus *and *A*. *sawadwongporni *were 15, 9, and 7 respectively. A total of 3,238 mosquitoes were processed – 1,655 mosquitoes (1,057 *A. dirus*, 402 *A. minimus *and 196 *A. sawadwongporni*) for round stages and ookinetes and 1,583 mosquitoes (1,423 *A. dirus*, 126 *A. minimus *and 34 *A. sawadwongporni*) for oocysts. A total of 37,421 parasites were counted – 428 gametocytes, 4,379 asexual blood stages, 11,786 round stages, 10,319 ookinetes, and 10,509 oocysts. For studies on late sporogony, the number of infections for *A. dirus*, and *A. minimus *were 5 and 2, respectively. Infections with *A*. *sawadwongporni *failed. A total of 514 mosquitoes (394 *A. dirus *and 120 *A. minimus*) were processed. A total of 41,212 parasites were counted – 13,123 oocysts, 14,693 haemocoel sporozoites, and 13,396 salivary gland sporozoites. Sporozoite count data were haemocytometer counts (*i.e.*, subsample counts), whereas round stage, ookinete and oocyst count data were direct counts.

### Early sporogony – volunteer infectiousness

Fourteen volunteers were infected with the VK210 strain of *P. vivax*, and one volunteer (Volunteer 3) was infected with the VK247 strain of *P. vivax*. All were symptomatic for malaria and displayed a range of haematocrits (32 to 54 %; mean = 43 ± 2 %), asexual parasitaemias (543 to 39,647 trophozoites per μl; mean = 5,552 ± 2,540), gametocytaemias (65 to 3,632 gametocytes per μl; mean = 540 ± 230) and female-to-male gametocyte ratios (0.5 to 6.1; mean = 2.7 ± 0.5). However, none of these parameters correlated with the infectiousness of volunteers to mosquitoes (*i.e*., oocyst density or prevalence; linear regressions, p > 0.05). Volunteers were classified retrospectively into 2 groups; HIGH (10 volunteers who at the time of donating blood, would likely perpetuate transmission in nature [*i.e*., volunteers 1, 2, 3, 5, 6, 11, 12, 13, 14, 15]) and LOW (five volunteers who were unlikely to perpetuate transmission in nature [*i.e*., volunteers 4, 7, 8, 9, 10]). The criterion for this classification was whether or not the volunteer yielded a geometric mean of ≥ 1 oocyst per midgut and ≥ 30% oocyst infection prevalence in *A. dirus*. In general, this classification held up for the two other mosquito species with the exception that three of the 10 volunteers classified as HIGH (*i.e*., volunteers 12, 13, 14) produced geometric means of less than 1 oocyst per midgut in *A. minimus *and *A. sawadwongporni*.

### Early sporogony – bloodmeal size, kinetics of erythrocyte digestion and ookinete formation

**Figure 2:**
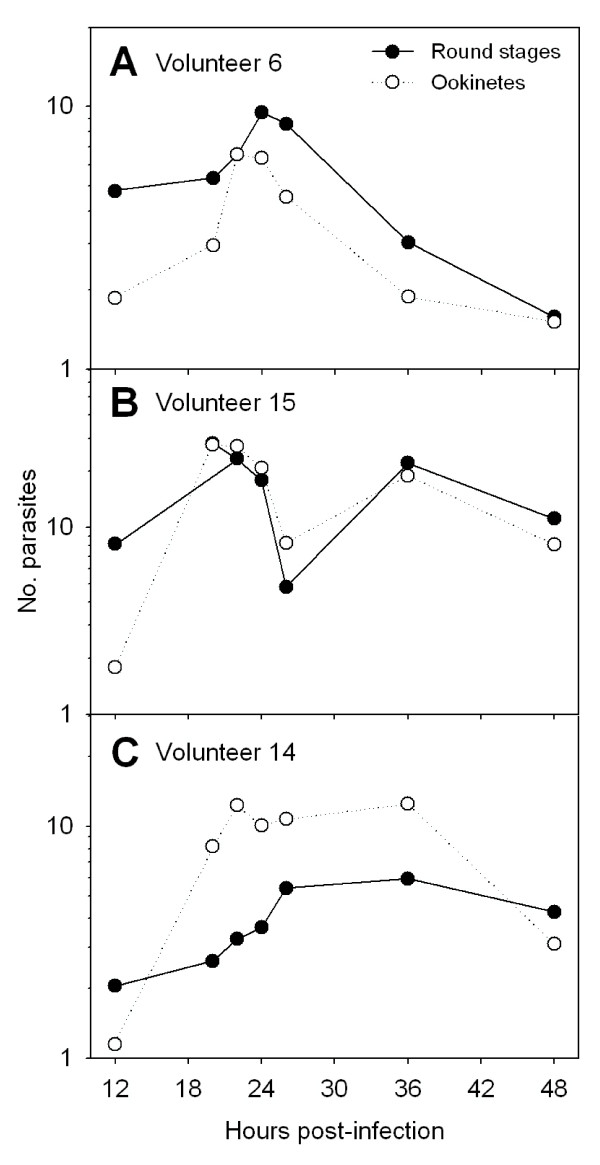
**Developmental kinetics of *Plasmodium vivax *round stage and ookinete life-stages in *Anopheles dirus *mosquitoes**. These figures show different patterns of ookinete development observed for individual infections and are representative of patterns observed for 31 separate infections in *A. dirus*, *A. minimus *and *A. sawadwongporni *mosquitoes.

Mean bloodmeal weights for *A*. *dirus*, *A*. *minimus *and *A*. *sawadwongporni *feeding on membrane feeders were 1.53 ± 0.42, 0.60 ± 0.27, and 0.64 ± 0.04 mg, respectively. There was a lag time of at least 12 (*A. sawadwongporni*) to 24 hours (*A. dirus*) before substantial digestion of erythrocytes occurred (data not shown). During this lag time, bloodmeals clotted and peritrophic matrices formed. Between 30 and 48 hours, digestion of erythrocytes accelerated and many erythrocytes appeared visibly damaged. By 48 hours, most of the erythrocytes were digested. Round stages and ookinetes were present in mosquito bloodmeals from 12 to 48 hours after ingestion of gametocytes. Developmental kinetics of these life-stages was asynchronous and there was considerable overlap in round stage and ookinete abundances. Although patterns varied among infections and mosquito species, 3 general patterns were observed; a single peak density (Figure 2A), a bimodal peak (Figure 2B) and a broad, extended peak (Figure 2C). There were no obvious trends in these patterns among mosquito species and no one pattern predominated. When all infections and mosquito species were combined, round stages were considerably more prevalent than ookinetes at 12 hours (81% versus 19%, respectively), but less so at 18 hours (58% versus 42%) and later (Table 1). This suggests that many round stages transformed to ookinetes between 12 and 18 hours. Retort-form ookinetes (Stages II and III, see Figure 1) were neither abundant nor prevalent during the entire sampling period, which suggested that these stages were ephemeral and developed rapidly into mature forms. Surprisingly, both round stages and ookinetes were still plentiful in mosquito midguts at 48 hours (Table 1, Figure 2), after most of the erythrocytes had been digested.

**Table 1:** Relative frequencies of *Plasmodium vivax *life-stages developing over 48 hours within *Anopheles *mosquitoes. Data for *A. dirus*, *A. minimus*, and *A. sawadwongporni *have been pooled.

TIME (hr)	Round stage	Stage II Ookinete	Stage III Ookinete	Stage IV-VI Ookinete	Total Number of Parasites
12	0.81	0.11	0.05	0.03	1,116
18	0.58	0.06	0.02	0.34	811
20	0.62	0.07	0.03	0.28	2,006
22	0.49	0.08	0.03	0.40	6,621
24	0.46	0.09	0.03	0.42	5,156
26	0.62	0.10	0.05	0.23	3,759
36	0.48	0.09	0.08	0.35	2,138
48	0.46	0.09	0.07	0.38	498

### Early sporogony – life-stage prevalences among mosquitoes

A high proportion of bloodmeals from mosquitoes fed on the HIGH category of volunteers contained round stages (>88%) and mature ookinetes (>75%), whereas a substantially lower proportion of bloodmeals from mosquitoes fed on the LOW category of volunteers contained round stages (<30%) and mature ookinetes (<13%) (Table 2). The composite age-class (*i.e.*, round stages and ookinetes combined) was comprised of a significantly greater proportion of round stages in the LOW category (94% of the total 1,426 parasites counted) than in the HIGH category (60% of the total 20,679 parasites counted) (Chi square value = 906, df = 1, *p *< 0.05). This indicates that gametogenesis and/or fertilization was a less frequent event in mosquitoes fed on the LOW category volunteers than in mosquitoes fed on HIGH category volunteers. Whether the lower frequency of round stages in the LOW category was due to poor gametogenesis or poor fertilization is unknown because 1) early development is not synchronous, and 2) it has never been firmly established that the monoclonal antibody used to detect pre-oocyst stages (*i.e*., Pvs25) can unequivocally differentiate round stages as either unfertilized female gametes or zygotes.

**Table 2:** Infection prevalences for *Plasmodium vivax *early sporogonic life-stages developing within *Anopheles *mosquitoes. The total numbers of mosquitoes examined per life-stage are given in parentheses. HIGH = volunteers who, at the time of donating blood, would be most likely to perpetuate *P. vivax *transmission in nature (*i.e.*, yielding ≥ 1 oocyst per mosquito in *A. dirus*). LOW = volunteer blood that would not be likely to perpetuate transmission in nature (*i.e.*, yielding < 1 oocyst per mosquito in *A. dirus*).

Mosquito Species	Number of Volunteers	Round stage	Ookinete	Oocyst
	HIGH			
*A. dirus*	10	88.1% (637)	78.6% (637)	53.8% (513)
*A. minimus*	5	89.3% (150)	78.0% (150)	56.4% (55)
*A. sawadwongporni*	6	90.4% (167)	81.4% (167)	29.4% (34)
	LOW			
*A. dirus*	5	21.7% (420)	7.4% (420)	3.2% (910)
*A. minimus*	4	28.2% (252)	12.7% (252)	2.8% (71)
*A. sawadwongporni*	1	3.4% (29)	0.0% (29)	Not determined

### Early sporogony – parasite mortality and cohort life tables

The average total parasite mortality (*K*) from macrogametocyte to oocyst for all infections and mosquito species combined was 1.83 (antilog = 68-fold or 98% loss) and ranged from 0.77 (6-fold or 83% loss) to 3.36 (2,291-fold or >99.9% loss) (Table 3). There was no overall effect on *K *due to the mosquito species in which parasites developed (ANOVA, F = 0.06, df = 2,24, p = 0.94), but there was a significant effect on *K *due to the particular volunteer from which the parasites originated (ANOVA, F = 4.65, df = 14, 24, p = 0.01). The average *k-1 *mortality for all infections combined was 1.27 (*i.e*., 19-fold or 95% loss) and ranged from 0.35 (2-fold or 55% loss) to 2.48 (302-fold or 99.7% loss). There was no significant effect on *k-1 *due to mosquito species (ANOVA, F = 1.97, df = 2,27, p = 0.16), but there was a significant effect due to volunteer (ANOVA, F = 5.16, df = 12, 27, p = 0.002). The average *k-2 *mortality for all infections combined was 0.38 (*i.e*., 2-fold or 57% loss) and ranged from 0 (no loss) to 1.96 (91-fold or 99% loss). There were no significant effects on *k-2 *due to either mosquito species (Kruskal-Wallis ANOVA, F = 0.06, df = 2,30, p = 0.94) or volunteer (Kruskal-Wallis ANOVA, F = 1.84, df = 14, 30, p = 0.12). The average *k-3 *mortality for all infections combined was 0.35 (*i.e*., 2-fold or 55% loss) and ranged from 0 (no loss) to 1.40 (25-fold or 96% loss). There were no significant effects on *k-3 *mortalities due to either mosquito species (Kruskal-Wallis ANOVA, F = 0.83, df = 2, 24, p = 0.45) or volunteer (Kruskal-Wallis ANOVA, F = 1.89, df = 14, 24, p = 0.16). Different volunteers had a highly significant effect on infection outcome and not all mosquito species fed on all the volunteers, so it was appropriate that only co-infections were used to compare population dynamics of sporogony among mosquito species. There were seven co-infections of *A. dirus *and *A. minimus *(Volunteers 7, 8, 9, 12, 13, 14, 15) having complete life tables, but only three co-infections with *A. sawadwongporni *having complete life tables (Volunteers 12, 14, 15) – too few to provide meaningful analyses of *k-2 *and *K *mortalities among *A. sawadwongporni *co-infections. Macrogametocyte densities were significantly greater in *A. dirus *than in co-infected *A. minimus *(geometric means = 190 and 75, respectively; Wilcoxon signed rank test, p = 0.008), presumably because *A. dirus *ingested more blood than *A. minimus *(see above). There was no significant difference between co-infected *A*. *dirus *and *A*. *minimus *with respect to densities of round stages (means = 15 and 14, respectively; paired t-test t = 0.09, df = 6, p = 0.93) or ookinetes (means = 9 and 5, respectively; paired t-test t = 1.29, df = 6, p = 0.24) but there was a significant difference with respect to oocyst densities (geometric means = 5 and 1, respectively; Wilcoxon signed rank test, p = 0.03). Although total parasite mortality did not differ significantly between co-infected *A. dirus *and *A*. *minimus *(*K *= 1.81 and 1.76, respectively; paired t-test t = 0.35, df = 6, p = 0.74), the partitioned mortalities indicated that population processes differed between *P. vivax *developing within the two mosquito species. Macrogametocyte mortality was significantly greater in *A*. *dirus *than in co-infected *A. minimus *(average *k-1*'s = 1.38 and 0.93, respectively; paired t-test t = 5.32, df = 6, p = 0.003). There was no significant difference in the mortality of round stages in either *A. dirus *or *A. minimus *(average *k-2*'s = 0.47 and 0.43, respectively; paired t-test, t = 0.25, df = 6; p = 0.81). However, ookinete mortality was significantly less in *A. dirus *than in *A*. *minimus *(average *k-3*'s = 0.15 and 0.40, respectively; paired t-test, t = 3.06, df = 6; p = 0.02). Thus, the patterns of *P. vivax *stage-specific mortality differed between co-infected *A*. *dirus *and *A*. *minimus*. Within *A*. *dirus*, *k-1 *mortality was significantly greater than either *k-2 *and *k-3 *mortalities (ANOVA with Tukey's post-hoc test, F = 14.8, df = 2,19, p = 0.0002), indicative of Type III survivorship [30]; whereas in *A*. *minimus *there were no significant differences between *k-1 *or *k-2 *or *k-3 *mortalities (ANOVA, F = 3.10, df = 2,18, p = 0.069), indicative of Type II survivorship.

**Table 3:** Cohort life tables for *Plasmodium vivax *developing within *Anopheles *mosquitoes. Stage-specific densities and mortality coefficients (*k*-values) for each cohort are given, where:*k*-1 = log_10_(macrogametocyte) - log_10_(round stage);*k*-2 = log_10_(round stage) - log_10_(ookinete);*k*-3 = log_10_(ookinete) - log_10_(oocyst);*K *=*k*-1 +*k*-2 +*k*-3. Values in parentheses = antilogs of averaged *k*-values. For each *Anopheles *species, parasite values are ranked in descending order of macrogametocyte densities within the HIGH and LOW categories of infection intensity.

Vol.	Class	Macrogametocyte	*k-1*	Round stage	*k-2*	Ookinete	*k-3*	Oocyst	*K*
	*Anopheles dirus*								

11	HIGH	2978.0	*	3.23	1.96	32.69	1.40	1.30	3.36
15		1242.0	1.63	29.27	0.00	31.37	0.00	59.50	1.63
2		812.9	1.12	61.05	0.21	37.69	0.35	17.00	1.68
5		416.3	*	42.39	0.33	192.58	0.65	42.70	0.98
6		358.6	1.38	15.08	0.17	10.15	0.63	2.40	2.18
1		332.8	1.44	12.20	0.00	13.43	0.00	25.10	1.44
13		277.7	0.85	39.33	0.52	11.88	0.77	2.00	2.14
3		233.3	1.37	10.02	0.00	14.03	0.00	85.80	1.37
14		96.1	*	6.71	0.77	16.20	0.00	61.70	0.77
12		56.6	1.20	3.54	0.08	2.96	0.00	10.60	1.28
8	LOW	804.4	2.15	5.70	0.77	0.15	0.06	0.00	2.98
7		165.5	0.93	19.63	1.17	1.32	0.25	0.30	2.35
10		138.8	2.06	0.23	0.09	0.00	0.00	0.10	2.15
4		101.8	1.58	2.70	0.34	1.23	0.14	0.60	2.06
9		34.7	1.54	0.00	0.00	0.00	0.00	0.00	1.54

Average Losses		1.44 (27-fold)		0.43 (3-fold)		0.28 (2-fold)		1.86 (72-fold)	

	*Anopheles minimus*								

15	HIGH	485.9	1.57	12.96	0.18	8.57	0.00	10.7	1.75
2		318.0	0.97	33.91	0.34	15.48	ND	ND†	ND
13		108.6	0.43	40.54	0.67	8.66	1.34	0.4	2.44
14		37.6	0.67	8.06	0.00	11.17	0.40	4.4	1.07
12		22.2	0.83	3.32	0.52	1.00	0.19	0.3	1.54
8	LOW	314.7	1.60	7.89	0.70	0.76	0.25	0	2.55
7		64.8	0.35	29.18	0.83	4.32	0.64	0	1.82
10		54.3	1.73	0.00	0.00	0.00	ND	ND	ND
9		13.6	1.03	0.37	0.14	0.00	0.00	0	1.17

Average Losses		1.02 (10 -fold)		0.33 (2-fold)		0.40 (2-fold)		1.76 (58-fold)	

	*Anopheles sawadwongporni*								

15	HIGH	518.3	1.51	15.92	0.34	7.26	0.58	1.9	2.43
2		339.2	1.00	34.23	0.54	9.98	ND	ND	ND
13		115.9	1.35	5.13	0.56	1.42	ND	ND	ND
3		97.4	1.08	8.08	0.24	4.67	ND	ND	ND
14		40.1	0.67	8.61	0.04	7.82	1.05	0.7	1.76
12		23.6	1.15	0.76	0.18	0.15	0.06	0	1.39
8	LOW	335.7	2.48	0.12	0.05	0.00	ND -	ND	ND

Average Losses		1.27 (19-fold)		0.38 (2-fold)		0.35 (2-fold)		1.83 (68-fold)	

* Not calculated because round stages were obviously under-sampled (note corresponding ookinete densities). Corresponding *k-2 *mortalities were therefore calculated as log_10_(macrogametocyte) – log_10 _(ookinete).

† ND = not determined due to insufficient numbers of mosquitoes available.

### Early sporogony – life-stage correlations

**Figure 3:**
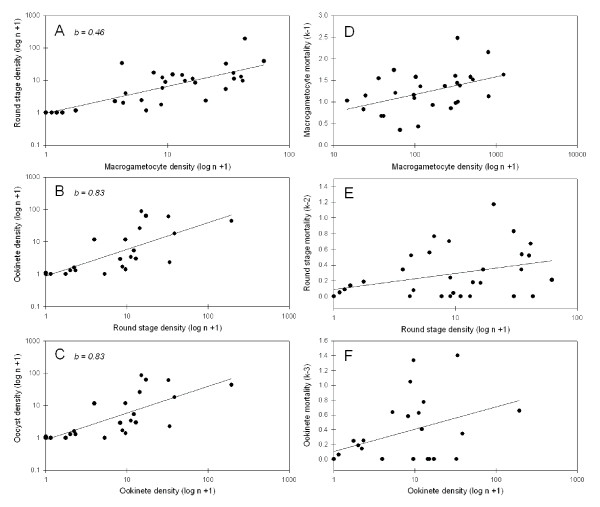
**Density relationships during *Plasmodium vivax *early sporogony in *Anopheles *mosquitoes**. Panels A, B, C illustrate density relationships between successive life-stages. Panel D, E, F illustrate density relationship between a life-stage and its corresponding mortality. Data for *A. dirus*, *A. minimus *and *A. sawadwongporni *mosquitoes were pooled because the regression equations for each of these relationships did not differ among mosquito species.

For each density relationship examined (*e.g*., ookinete vs. oocyst densities, *etc*.) there were no significant differences among the 3 mosquito species with respect to the slopes or elevations of individual regression equations (analyses of covariance, p < 0.05 [31]). Therefore, data were pooled among mosquito species. There was no correlation between macrogametocyte density and oocyst prevalence (r^2 ^= 0.11, F = 2.9, df = 1,24, p = 0.10), indicating that oocyst infection prevalence could not be predicted based on examination of a blood smear. However, the densities of successive life-stages were all significantly correlated – *i.e*., macrogametocyte and round stage densities (Figure 3A, r^2 ^= 0.23, F = 8.3, df = 1,30, p = 0.007), round stage and ookinete densities (Figure 3B, r^2 ^= 0.58, F = 39.6, df = 1,30, p < 0.0001), and ookinete and oocyst densities (Figure 3C, r^2 ^= 0.55, F = 28.0, df = 1,24, p < 0.0001). Likewise, the correlations between life-stage densities and the stage-specific mortalities (*k*'s) acting on those life-stages were also statistically significant (or nearly so) – *i.e*., macrogametocyte density and *k-1 *(Figure 3D, r^2 ^= 0.28, F = 11.3, df = 1,30, p = 0.002), round stage density and *k-2 *(Figure 3E, r^2 ^= 0.12, F = 4.1, df = 1,30, p = 0.053), ookinete density and *k-3 *(Figure 3F, r^2 ^= 0.18, F = 5.0, df = 1,24, p = 0.036). A significant positive relationship between life-stage density and its corresponding *k*-value is suggestive of density-dependant mortality. However these variables are not independent of one another and, therefore, suspected density dependence could be spurious due to sampling errors [26]. To validate density dependence, the regression equations for successive life-stage densities (Figure 3A–C) were re-examined to test whether regression slopes departed significantly from 1.0 [32], which would confirm that density dependence is real [26]. The only density relationship whose slope departed significantly from 1.0 was that of macrogametocyte and round stage densities (Figure 3A, slope = 0.46, t-test, t = 2.04, df = 29, p < 0.05). This indicates that density dependent mortality occurred during the macrogametocyte to round stage transition (*i.e*., gametogenesis/fertilization) but not during other life-stage transitions of early sporogony.

### Early sporogony – parasite distribution

**Figure 4:**
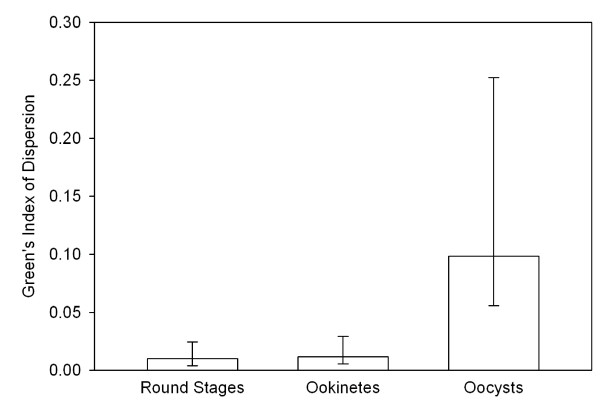
**Dispersal indices for *Plasmodium vivax *life-stages developing within *Anopheles dirus*, *A. minimus *and *A. sawadwongporni *mosquitoes**. Bars indicate the mean indices for each parasite life-stage. Error bars indicate 95% confidence limits. A Green's index of zero indicates that parasites were randomly distributed among mosquitoes. Higher values indicate that parasites were more unevenly distributed among mosquitoes, suggesting that there was greater heterogeneity among individual mosquitoes in their susceptibility for that specific parasite life-stage.

Patterns of parasite distribution within *A. dirus, A. minimus *and *A. sawadwongporni *were described by calculating Green's index of dispersion for round stages, ookinetes and oocysts in each infection where more than one infected mosquito was found for each of these life-stages (Figure 4). Mean dispersion indices for round stages (0.0102) and ookinetes (0.0119) did not differ significantly from one another (paired t-test, t = 0.60, df = 17, p = 0.56) but both indices were significantly lower than mean dispersion indices for oocysts (0.0984) (paired t-tests, t values>3.0, df = 17, p values = 0.006). Round stages and ookinetes developed within mosquitoes in a more homogenous manner and were more randomly distributed among individual mosquitoes than were oocysts – regardless of parasite density, fertilization success or parasite origin (HIGH versus LOW volunteer).

### Late sporogony – kinetics of oocyst growth, sporozoite release and invasion into mosquito salivary glands

**Figure 5:**
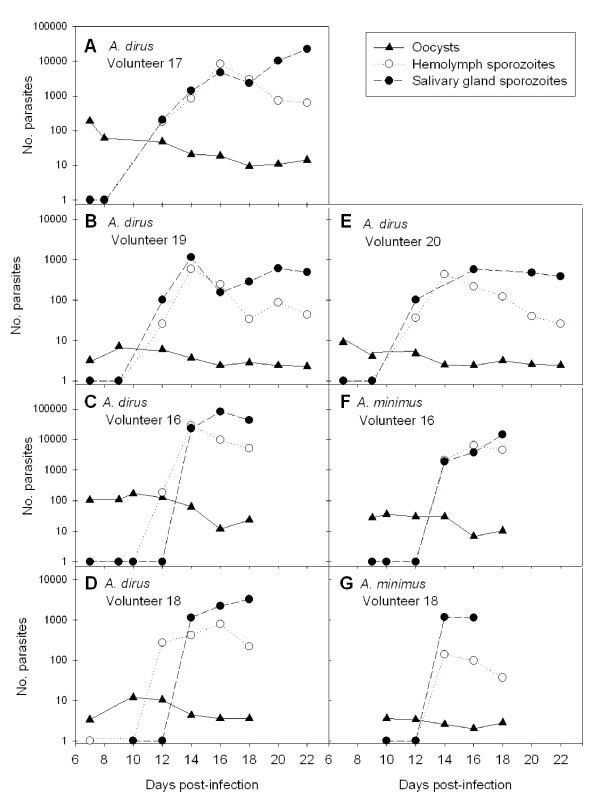
**Developmental kinetics of *Plasmodium vivax *sporozoite release from oocysts and invasion into mosquito salivary glands**. Parasite densities (n+1; shown on log scale) were recorded for *Anopheles dirus *(Panels A-E) and *A. minimus *(Panels F-G) from Days 7 to 22 post-infection (p.i.). Results for co-infections of *A. dirus *and *A. minimus *are shown for Volunteers 16 (Panels C and F) and 18 (Panels D and G); data were collected only until Day 18 p.i.

All five volunteers providing parasites for this phase of the study were infected with the VK210 strain of *P. vivax*. There was no significant difference in the diameters of oocysts developing within *A. dirus *versus *A. minimu*s (t-test, t = -0.01, d = 316, p = 0.99). Mean oocyst diameters did not increase significantly from days 7–10, but increased almost 2-fold from days 12 to 14. Thereafter, oocyst size stabilized and mean oocyst diameters did not change significantly throughout the remainder of the study (Table 4). The rapid swelling of oocysts at days 12–14 corresponded to sporozoite release (Figure 5). Oocyst release of sporozoites into the haemocoel occurred as early as day 12 for *A. dirus *and day 14 for *A. minimus*. Sporozoite invasion into the salivary glands occurred at day 14 for both species. This indicated that sporozoite invasion into the glands of *A*. *dirus *may have been a somewhat more protracted process requiring one to two days, whereas in *A. minimus*, sporozoites invaded the salivary glands within one day after being released from the oocyst. The kinetics of sporozoite release and invasion into the salivary glands were similar for each mosquito species regardless of whether the infection was of high or low intensity (Figure 5, Volunteers 16 and 18, respectively). However, not all oocysts released sporozoites. Even after massive release of sporozoites into the haemocoel on days 12 and 14, there remained a substantial number of fully-formed oocysts on the gut that had failed to release sporozoites by day 22 (Figure 5, Volunteers 17, 19 and 20).

**Table 4:** *Plasmodium vivax *oocyst growth in *Anopheles dirus *and *A. minimus *mosquitoes during late sporogony. Values indicate mean oocyst diameters (μm) with 95% confidence intervals in parentheses and accompanying letters signifying statistically significant differences in mean diameters.

Day	7	9	10	12	14	16	18	20	22
Oocyst Diameter (μm)	27 c (22–32)	31 c (27–35)	34 c (31–36)	49 b (46–51)	58 a (55–61)	58 a (55–62)	55 ab (52–58)	53 ab (46–60)	52 ab (42–63)
No. Mosq.	13	12	28	58	64	57	47	24	15

*Different letters indicate significant differences in oocyst diameter (Tukey post-hoc test, p < 0.05).

### Late sporogony – sporozoite production and invasion efficiency into mosquito salivary glands

The per capita production of sporozoites was calculated for each infection using mean oocyst densities from mosquitoes sampled on days 7 to 10 prior to the release of sporozoites. Mean oocyst densities ranged from two to 131 oocysts per mosquito (Table 5). Total sporozoite production was calculated using mosquitoes sampled on days 12 to 18 and included all infected mosquitoes, even those that were oocyst-positive but sporozoite-negative (i.e., to account for possible abortive infections). Mean per capita production of sporozoites ranged from 169 to 784 sporozoites per oocyst. There was no significant difference in sporozoite production between oocysts developing within *A. dirus *versus those within *A. minimu*s (t-test, t = -1.87, d = 5, p = 0.12). The overall average for both mosquito species combined was 508 ± 230 sporozoites per oocyst. Only sporozoite-positive mosquitoes were used to calculate the efficiency of sporozoite invasion into the salivary glands. Sporozoite invasion into salivary glands was consistently efficient from infection to infection, with no significant difference between sporozoites developing within *A*. *dirus *versus *A*. *minimus *(t-test, t = 0.46, d = 5, p = 0.66). The overall proportion of haemocoel sporozoites that succeeded in invading the salivary glands was estimated to be 0.737 ± 0.099, or roughly three-quarters of the total sporozoites produced.

**Table 5:** Per capita production of *Plasmodium vivax *sporozoites and subsequent invasion efficiency into mosquito salivary glands.

Volunteer	Geometric Mean Oocyst Density (days 7–12)	Sporozoites Produced per Oocyst (days 12–18)	Proportion of Sporozoites Invading the Salivary Glands (days 12–18)
	*Anopheles dirus*		
	
17	42	310	0.613
18	5	668	0.791
19	2	350	0.742
20	3	169	0.840
Average ± SD		422 ± 212	0.749 ± 0.085

	*Anopheles minimus*		
	
16	30	664	0.590
18	2	784	0.826
Average ± SD		724 ± 85	0.708 ± 0.167

Overall average sporozoite production for both mosquito species combined = 508 ± 230 sporozoite/oocyst. Overall proportion of sporozoites entering salivary glands = 0.737 ± 0.099.

### Late sporogony – life-stage correlations

**Figure 6:**
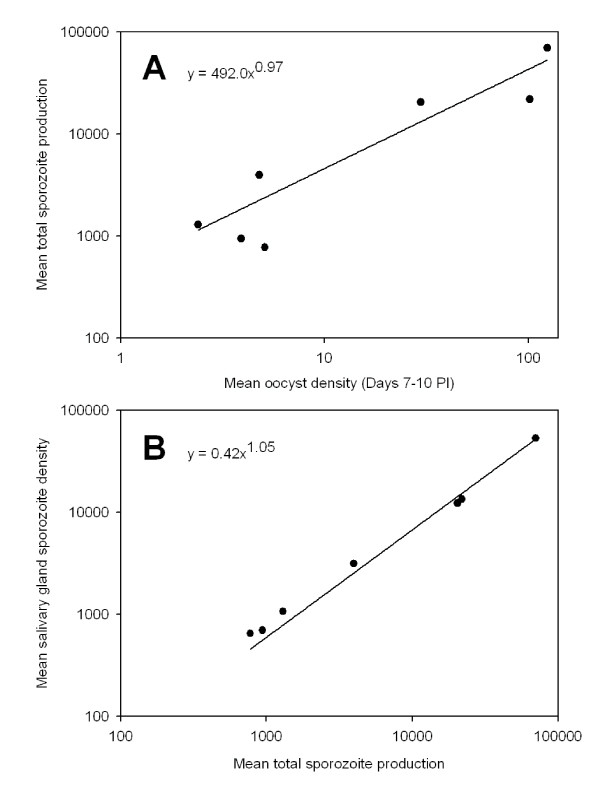
**Density relationships of *Plasmodium vivax *during late sporogony in *Anopheles dirus *and *A. minimus *mosquitoes**. A) *Plasmodium vivax *oocyst density (Days 7–10) and sporozoite production (Days 12–18) and B) sporozoite production and density of sporozoites in the mosquito salivary glands (Days 12–18).

There were no differences in regression equations between *A*. *dirus *and *A*. *minimus *(analyses of covariance, p's > 0.05) and therefore data were pooled. There was a significant correlation between the average oocyst density present on midguts prior to sporozoite release and the subsequent production of sporozoites (Figure 6A; r^2 ^= 0.86, F= 31.9, df= 1,6, p= 0.002). Likewise, there was a highly significant correlation between average sporozoite production and sporozoite density in the salivary glands (Figure 6B; r^2 ^= 0.99, F = 1226.6, df = 1,6, p < 0.0001).

## Discussion

It has long been known that some gametocyte carriers are more infectious to mosquitoes and produce more oocysts than do other gametocyte carriers [33-35]. This was also the case in the present study. On average, *P. vivax *populations experienced a 68-fold loss in abundance from macrogametocyte to oocyst and there were no differences in the magnitude of overall parasite losses among the 3 mosquito species tested (*K*, Table 3). However, losses within individual cohort infections varied tremendously (6-fold to over 2,000-fold) depending on the source of the gametocyte population (i.e., the volunteer). Life tables showed clearly that mortality during gametogenesis and fertilization (*i.e*., *k-1*) was generally the most critical transition determining an infection outcome (Table 3). Importantly, differences among volunteers in gametocyte and trophozoite densities, gametocyte sex ratios and blood haematocrits did not correlate with differences in volunteer infectiousness – an observation noted previously by other workers [36-38]. Obviously, something else influenced the early developmental success rates of different gametocyte populations. In the broad sense, possibilities may include factors intrinsic to the parasite (*e.g*., gametocyte immaturity or senescence) or extrinsic factors contained within the blood (*e.g*., antibody, cytokines, drugs, etc.). However, it seems unlikely that mosquito factors contributed to the high infection-to-infection variability observed in *k-1 *(macrogametocyte) or *k-2 *(round stage) mortalities because the dispersion indices for round stages and ookinetes were essentially zero (Figure 4). This means that within almost every infection, the processes affecting production of round stages and ookinetes occurred randomly among mosquitoes, with little mosquito-to-mosquito heterogeneity – regardless of whether a particular infection was successful or not. Furthermore, significant variability in *k-1 *mortalities was observed among volunteers but not among mosquito species. Thus, blood and/or parasite factors exerted a more dominant influence on *k-1 *and *k-2 *mortalities than did mosquito factors.

One source of *k-1 *mortality may be inferred by examining differences among individual infections in life-stage prevalence (Table 2). In this study, all unfed mosquitoes were removed at the onset of each infection so that in theory the starting gametocyte prevalence was virtually 100%. However by 12 to 48 h after ingesting gametocytes, a significantly lower prevalence of round stages was observed in mosquitoes fed blood from poorly infectious people (LOW; Table 2) than in mosquitoes fed blood from more infectious people (HIGH). This marked decline in the prevalence of round stages suggests that gametogenesis and/or fertilization was sub-optimal in blood from poorly infectious people. In those mosquitoes that did produce round stages when fed poorly infectious blood, the ratio of round stages to mature ookinetes was significantly higher than in mosquitoes fed more infectious blood. Presumably, a higher ratio of round stages-to-ookinetes persisting throughout the 2 day sampling period means that round stages produced from the poorly infectious volunteers were less likely to complete their development to ookinetes. Thus, *P. vivax *gametocytes within poorly infectious people displayed sub-optimal fertilization and/or zygote differentiation. Another intriguing, albeit minor, source of *k-1 *mortality was that of density-dependant mortality of macrogametocytes – *i.e*., the per capita conversion of gametocytes to round stages decreased with increasing gametocyte density (Figures 3A and 3D). The mechanisms underlying this form of population regulation is speculative but one plausible explanation is polyspermy – *i.e*., simultaneous fertilization of a single female gamete with more than one male gamete. As the density of gametocytes increases, the likelihood of polyspermy would also increase. Polyspermy is lethal to the zygotes of most organisms [39] but it has only been described in eukaryotic organisms. Density dependent mortality was not confirmed for later life-stages.

Overall, ookinete mortality was low (2-fold loss). Unlike that of gametocytes and round stages, mortality of ookinetes (*k-3*) was probably more strongly influenced by mosquito factors. In paired comparisons, dispersion indices for round stages and ookinetes were significantly lower than indices for oocysts (Figure 4). Thus, parasites within the blood meal were initially distributed randomly among mosquitoes but became more aggregated as ookinetes exited the midgut to form oocysts. The implication is that there was heterogeneity with respect to an individual mosquito's permissiveness to ookinete penetration and establishment on the outer midgut wall. Some mosquitoes simply presented a more hostile environment than others. Mosquito factors that act as mortality factors to block the ookinete conversion to oocyst include peritrophic matrix [40-42], digestive enzymes [43-45] and an array of immune effectors produced in response to ookinete invasion [reviewed in [46-48]]. It may be expected that there is some degree of variation in the vigor and timing of these processes among individual mosquitoes and that this variation, coupled with the individual variation in kinetics of ookinete formation (see Figure 2), produced the observed heterogeneity in oocyst densities. Interestingly, the mean dispersion indices for *P. vivax *oocysts recorded in this study using laboratory colonies of *A. dirus *(0.092 ± 0.138) and *A. minimus *(0.082 ± 0.093) mosquitoes were not statistically different from dispersion indices calculated from the published data of Rosenberg et al. [49] on *P. vivax *oocyst populations infecting wild-caught *A. dirus *(0.114) and *A. minimus *(0.087) from southeastern Thailand (t-tests, p > 0.58). This supports the notion that the population dynamics of early sporogony described herein are similar to that which occurs in nature.

The classic view of sporozoite production is that each oocyst contains several thousand sporozoites. Indeed, meticulous studies where individual *P. vivax *oocysts were plucked from the midguts of *A. dirus *reported that each of 26 oocysts contained a mean of 3,688 sporozoites [50]. However, the present studyclearly indicates that not every oocyst achieves its full production potential (Table 5, Figure 5). Some oocysts probably contribute more sporozoites to the overall standing crop than others, whereas some oocysts may not contribute any sporozoites at all. The estimates of 169 to 784 sporozoites per oocyst reflect the per capita production of the entire oocyst population on a mosquito midgut and are reasonably similar to estimates obtained by linear regression of oocyst densities plotted against salivary gland sporozoites – *i.e*., 850 gland sporozoites per oocyst for *P. vivax *in *A. dirus *[38] and 663 gland sporozoites per oocyst for *P. falciparum *in *A gambiae *[12]. In the present study, apparently healthy oocysts were present on midguts for up to 22 days, i.e., more than 1 week after the initial surge of sporozoites into the haemocoel (Figure 5). Why some oocysts failed to release sporozoites is not known but of the 23,632 oocysts examined during the course of this study, no melanized oocysts were observed, suggesting that the mosquito melanization response was not responsible. If each *P. vivax *oocyst in *A. dirus *contains an average of 3,688 sporozoites [50] but the per capita production of the oocyst population averaged only 422 sporozoites per oocyst (Table 5), then only 11% of the *potential *sporozoite production and release was actually realized by day 18 of infection. It remains to be determined whether laggard oocysts sequentially release fresh sporozoites into the haemocoel throughout the life time of their mosquito host or whether they simply stop producing sporozoites.

Even though initial oocyst release of sporozoites was inefficient, the invasion of *P. vivax *haemolymph sporozoites into the salivary glands of *A. dirus *and *A. minimus *was very efficient. Nearly 75% of all sporozoites produced successfully entered the glands (Table 5). These estimates are similar to efficiency estimates using similar methodology for *P. falciparum *sporozoite invasion into *A*. *gambiae *salivary glands (= 89%, [12]). Total sporozoite production was related linearly to oocyst density and likewise, sporozoite density in the salivary glands was linearly related to the total sporozoite production of an infection (Figure 6). Thus within the intensity levels observed in this study, there was no obvious "saturation effect" of having too many oocysts on the gut or too many sporozoites in the haemolymph or salivary glands. This finding is compatible with findings from other *Plasmodium*/*Anopheles *systems [12,51] and, when taken together, suggest that once ookinetes cross the midgut and establish themselves as oocysts, nutrients for further parasite population growth within mosquitoes are essentially unlimited and there is no "carrying capacity" imposed on developing oocysts by their "habitat".

## Conclusion

This study describes the population dynamics of sporogony for 20 natural isolates of *P. vivax *from western Thailand in three species of colonized *Anopheles *species; *A*. *dirus*, *A*. *minimus *and *A*. *sawadwongporni*. Overall, there was a 68-fold loss in abundance in parasite development from macrogametocyte to oocyst but the magnitude of parasite losses within individual infections ranged from 6-fold to over 2,000-fold. Gametogenesis and/or fertilization were the most critical processes determining the infection outcome. Subsequent parasite losses during round stage transformation and ookinete migration were generally less variable among infections. Indices of parasite dispersion suggested that parasites losses during fertilization and round stage transformation were more influenced by factors intrinsic to the parasite and/or factors within human blood, whereas losses during ookinete migration were more strongly influenced by mosquito factors. Sporozoite release from oocysts occurred on days 12 and 14 for *A*. *dirus *and *A*. *minimus*, respectively. Sporozoites in the haemocoel invaded the salivary glands efficiently (ca. 74%) within a day or two. Not all oocysts produced sporozoites. Understanding population dynamics of sporogony in nature may help predict the efficacy of intervention strategies that target sporogony.

## Authors' contributions

JV and RC designed the study. JS managed logistics and coordinated with the Thai Ministry of Public Health. GZ, NP, SP, GG and JV conducted the studies. GZ, JB, and JV performed statistical analyses and artwork. GZ and JV wrote the manuscript. All authors have read and approved the final manuscript.
